# Supplementary material for: Advanced Manufacturing in the Fabrication of a Lifelike Brain Glioblastoma Simulator for the Training of Neurosurgeons
Source: Polymers (Basel). 2022 Mar 8;14(6):1072. doi: 10.3390/polym14061072 (PMC8948645; doi:10.3390/polym14061072)
Supplement: Supplementary file 1 [file polymers-14-01072-s001.zip › polymers-1598205-supplementary.pdf]

(Supplementary Materials)
Advanced Manufacturing in the Fabrication of a Lifelike Brain Glioblastoma Simulator for the Training of Neurosurgeons

Pin-Chuan Chen [1,2,], Yu-Wen Yang[1], Jang-Chun Lin [3,4,**],and Wei-Hsiu Liu [5,6,*]**

[1] Department of Mechanical Engineering, National Taiwan University of Science and Technology, 106 Taipei, Taiwan; Pcchen@mail.ntust.edu.tw (P.-C.C.);lylidy@gmail.com (Y.-W.Y.)
[2] High Speed 3D Printing Research Center, National Taiwan University of Science and Technology, 106 Taipei, Taiwan
[3] Department of Radiation Oncology, Shuang Ho Hospital, Taipei Medical University, 106 Taipei, Taiwan; 13451@s.tmu.edu.tw
[4] Department of Radiology, School of Medicine, College of Medicine, Taipei Medical University, 106 Taipei, Taiwan
[5] Department of Neurological Surgery, Tri-Service General Hospital and National Defense Medical Center, 114 Taipei, Taiwan
[6] Department of Surgery, School of Medicine, National Defense Medical Center, 114 Taipei, Taiwan

***** Correspondence: doc20444@mail.ndmctsgh.edu.tw; Tel.: +886-2-87927177; Fax: +886-2-87927178
** Both authors have equal contribution

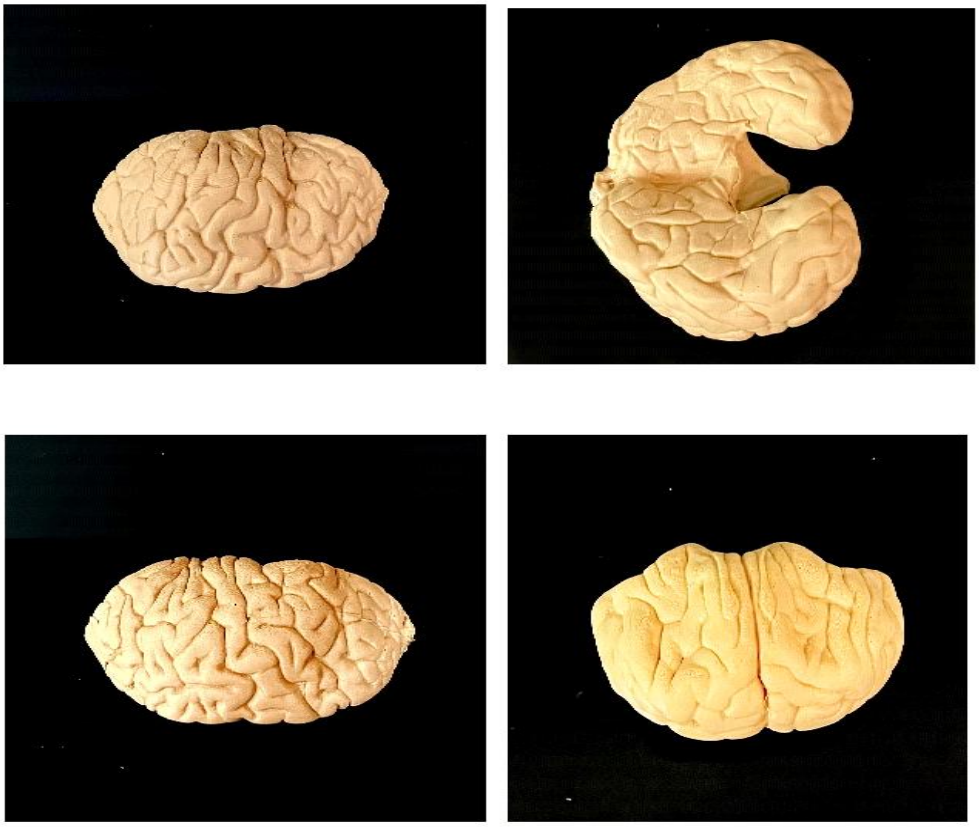

Figure S1: (a) frontal lobe; (b) temporal lobe; (c) occipital lobe; (d) parietal lobe;

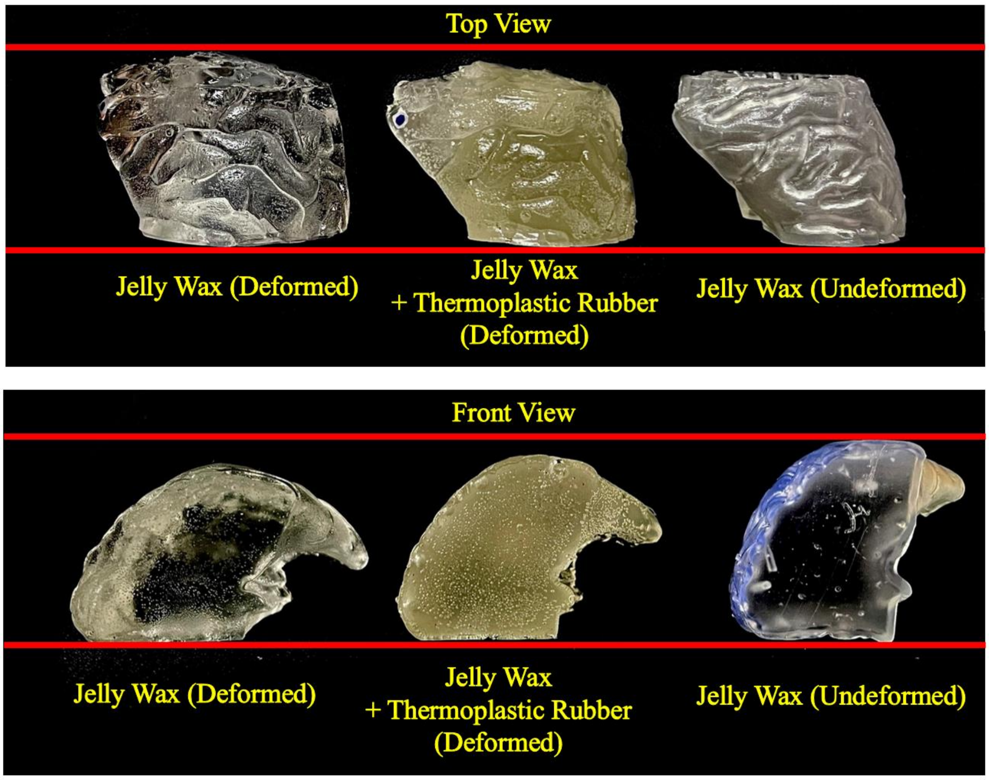

Figure S2: The deformation in terms of height and width of jelly wax and jelly wax plus thermoplastic rubber.

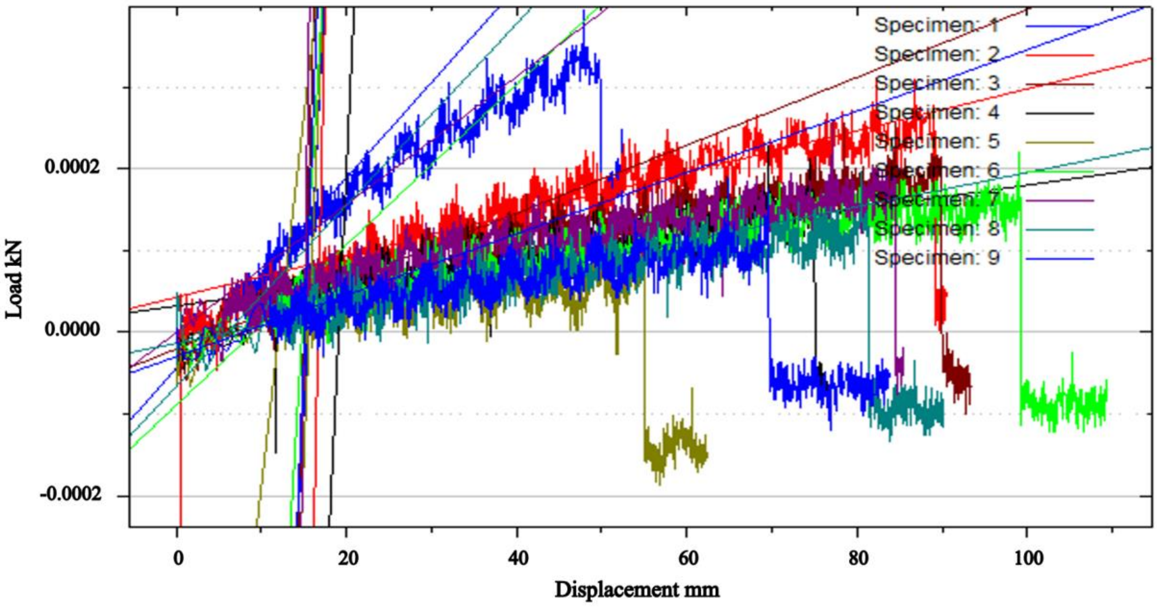

Figure S3: The maximum tensile test of jelly wax
